# Transcriptome-wide identification and expression profiles of the WRKY transcription factor family in Broomcorn millet (*Panicum miliaceum* L.)

**DOI:** 10.1186/s12864-016-2677-3

**Published:** 2016-05-10

**Authors:** Hong Yue, Meng Wang, Siyan Liu, Xianghong Du, Weining Song, Xiaojun Nie

**Affiliations:** College of Agronomy, Northwest A&F University, 3 Taicheng Road, Yangling, Shaanxi 712100 China; State Key Laboratory of Crop Stress Biology in Arid Areas, Yangling, Shaanxi 712100 China; Yangling Branch of China Wheat Improvement Center, Northwest A&F University, Yangling, Shaanxi 712100 China

**Keywords:** Abiotic stress, Broomcorn millet, qRT-PCR, WRKY

## Abstract

**Background:**

WRKY genes, as the most pivotal transcription factors in plants, play the indispensable roles in regulating various physiological processes, including plant growth and development as well as in response to stresses. Broomcorn millet is one of the most important crops in drought areas worldwide. However, the WRKY gene family in broomcorn millet remains unknown.

**Results:**

A total of 32 PmWRKY genes were identified in this study using computational prediction method. Structural analysis found that PmWRKY proteins contained a highly conserved motif WRKYGQK and two common variant motifs, namely WRKYGKK and WRKYGEK. Phylogenetic analysis of PmWRKYs together with the homologous genes from the representative species could classify them into three groups, with the number of 1, 15, and 16, respectively. Finally, the transcriptional profiles of these 32 PmWRKY genes in various tissues or under different abiotic stresses were systematically investigated using qRT-PCR analysis. Results showed that the expression level of 22 PmWRKY genes varied significantly under one or more abiotic stress treatments, which could be defined as abiotic stress-responsive genes.

**Conclusions:**

This was the first study to identify the organization and transcriptional profiles of PmWRKY genes, which not only facilitates the functional analysis of the PmWRKY genes, and also lays the foundation to reveal the molecular mechanism of stress tolerance in this important crop.

**Electronic supplementary material:**

The online version of this article (doi:10.1186/s12864-016-2677-3) contains supplementary material, which is available to authorized users.

## Background

Broomcorn millet (*Panicum miliaceum* L.) is one of the world’s oldest cultivated crops and also is one of the most important crops in drought areas. Before the domestication of rice and wheat, broomcorn millet was the main food staple in many semiarid regions of Asia, including China, Korea, India, Russia, Japan, and even in the entire Eurasian continent [1–3]. It’s supposed that broomcorn millet had been cultivated in East Asia since 10,000 years ago and played an essential impact on human civilisation [4]. Moreover, broomcorn millet has many favored agronomic traits, such as short growing season, high productivity, little nutrient, and water requirements as well as excellent tolerance to salt, drought, high temperature, and other extreme conditions.

With the advent of global climate changes, abiotic stresses such as salt, drought, freezing, and heat have become the hazardous threats to world’s agricultural production. Growth and development of crops were suppressed under abiotic stresses and then result in huge yield loss of production. It has been discovered that a large number of genes were induced when plants suffered to abiotic stresses, which could be were considered as stress-responsive genes. In general, the stress-responsive genes could be divided into functional proteins and regulatory factors [5]. Transcription factors, which are the dominant groups of regulatory factors to control the expression of other target genes, play the core roles in gene expression regulation and signal transduction.

WRKY transcription factors contain a highly conserved 60 amino acid DNA-binding regions, which designated as WRKY domain consisting of the conserved peptide sequence WRKYGQK (N-terminus) and a zinc-finger motif (C-terminus) [6]. According to the number of WRKY domains and structure features of zinc-finger motif, WRKY gene family can be classified into three groups. For the number of WRKY domain, group I of WRKY members contain two WRKY domains, while group II and group III have only one WRKY domain, respectively. For the structure features of zinc-finger motif, group I and group II have the C2H2 (C–X4–C–X22–23–HXH) zinc finger motif, while group III has the C2CH (C-X7-C-X23-HXC) motif. Furthermore, the Group II can be divided into five subgroups, including from IIa to IIe [7].

WRKY gene family has been revealed to be one of the most important transcription factor families in plant, which played the important roles in regulating various development and physiological processes [8]. In *Arabidopsis*, it was found that *AtWRKY75* has a negative effect on root hair development [9]. *AtWRKY18* and *AtWRKY53* have a positive effect on leaf senescence. In addition, over-expression of *AtWRKY18* and *AtWRKY53* could results in delaying senescence [10], and *AtWRKY34* is required for male gametogenesis [11]. It has also proven that WRKY proteins were involved in regulating pathogen infection and various abiotic stresses defenses [12–14]. Previous report has found that over-expression of *GhWRKY44* not only enhanced resistance to fungal pathogen *R. solani* in cotton, but also improved tolerance to bacterial pathogen *R. solanacearum* [15]. In terms of *GhWRKY39-1*, its expression was induced by pathogen infection and salt stresses [16]. Additionally, *TaWRKY10* gene was found to be played a crucial role in wheat under salinity, cold, and drought stress responses [17]. Furthermore, co-expression of WRKYs had capacity to form the network to resist the stress. For instance, over-expression of *OsWRKY11* and *OsWRKY45* genes improved drought tolerance in rice [18]. Over-expression of *GmWRKY54* and *GmWRKY13* improved salt tolerance in *Arabidopsis* [19]. At present, extensive studies have been performed to identify and dissect the function of WRKY gene family in different plant species, included *Arabidopsis* [20], rice [21], wheat [22], grape [23], maize [24], and *Brachypodium distachyon* [25]. However, the organization and function of *WRKY* genes in broomcorn millet is completely unknown.

Given the above arguments, this study was performed with the following four objectives: (1) to systematically explore the *WRKY* genes in Broomcorn millet using the available EST and unigene sequences; (2) to investigate the gene composition, protein structure and orthologs construction of these identified WRKY genes; (3) to elucidate the evolutionary relationship and classification among the WRKY genes in broomcorn millet; (4) to comprehensively investigate expression profiles of these PmWRKY genes in various tissues or abiotic stress response to identify appropriate candidates for further functional studies. Taken together, the study will provide the novel insight into protein structures, evolutionary relationships, and expression pattern of WRKYs in Broomcorn millet, which will facilitate for further investigation of the functions of PmWRKY genes.

## Result and discussion

### Identification of PmWRKY genes

As one of the most important transcription factors in plant, WRKY gene family play the pivotal role in regulating plant growth and development as well as stress response [8]. Although the functions of several WRKY genes in Arabidopsis and other model crops have been systematically studied [16–19], little is known about this gene family in the oldest food crops, broomcorn millet. At present, there is not any WRKY gene has been cloning or reported from broomcorn millet, which limited the study to reveal the molecular mechanism of stress resistance of this inimitable stress-tolerant crops.

To explore the organization and function of WRKY genes in broomcorn millet, the available ESTs and transcriptome contigs of this species were applied to identify WRKY genes through computational prediction. By blast search of the foxtail millet WRKY genes, a total of 59 unique ESTs/contigs showed high similarity (e-values range from 5.0 × 10^−23^ to 3.0 × 10^−108^). These contigs were considered as the putative WRKY genes in broomcorn millet. Then, the obtained sequences were submitted to the NCBI-CDD web server to analyze their conserved protein domain. Furthermore, the sequences contained the complete WRKY domain were further filtered to remove repeats by BLAST against foxtail millet transcription factor database (http://59.163.192.91/FmTFDb/index.html). Finally, a total of 32 unique PmWRKY genes includes complete conserved domain were identified in this study.

### Phylogenetic analysis of PmWRKY genes

**Fig. 1:**
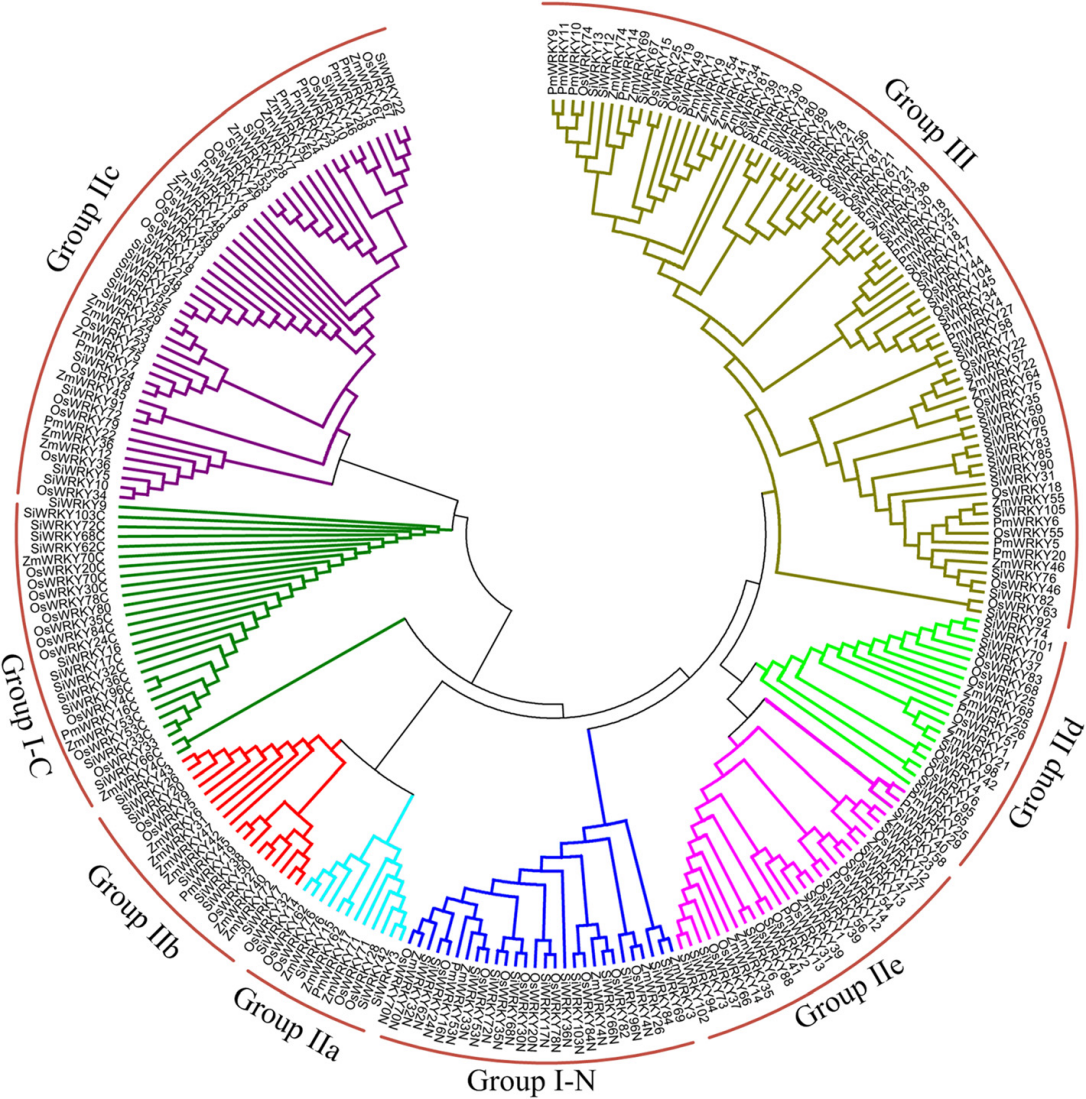
Phylogenetic analysis of WRKY proteins among Broomcorn millet (32), foxtail millet (103), rice (75) and maize (50)

The WRKY gene family, which are high conserved in both monocots and eudicots, could be divided into three groups according to the protein structure and sequence similarity [21, 24]. To categorize and investigate the evolutionary relationships of PmWRKY genes, all putative 32 PmWRKY proteins in broomcorn millet, 75 WRKY proteins in rice, 50 WRKY proteins in maize, and 103 WRKY proteins in foxtail millet were selected to perform phylogenetic analysis. As shown in Fig. 1, phylogenetic results revealed that these PmWRKY proteins could be categorized into three groups, including Group I, Group II, and Group III. *PmWRKY16* protein were considered as Group I, which included two WRKY domains and C2H2 (C–X4–C–X22–23–HXH) zinc-finger motif. A total of 15 PmWRKY proteins contained one WRKY domain and C2H2 (C-X4–5-C-X23-HXH) zinc-binding motif, which were classified to group II. The remaining 16 genes were assigned to group III, which had single WRKY domain and C2CH (C-X7-C-X23-HXC) zinc-binding motif. According to the WRKY subgroup classification of rice, maize, and foxtail millet, the group II of PmWRKYs was further classified into five subgroups, including group IIa (1), IIb (1) IIc (8), IId (1), and IIe (4).

**Fig. 2:**
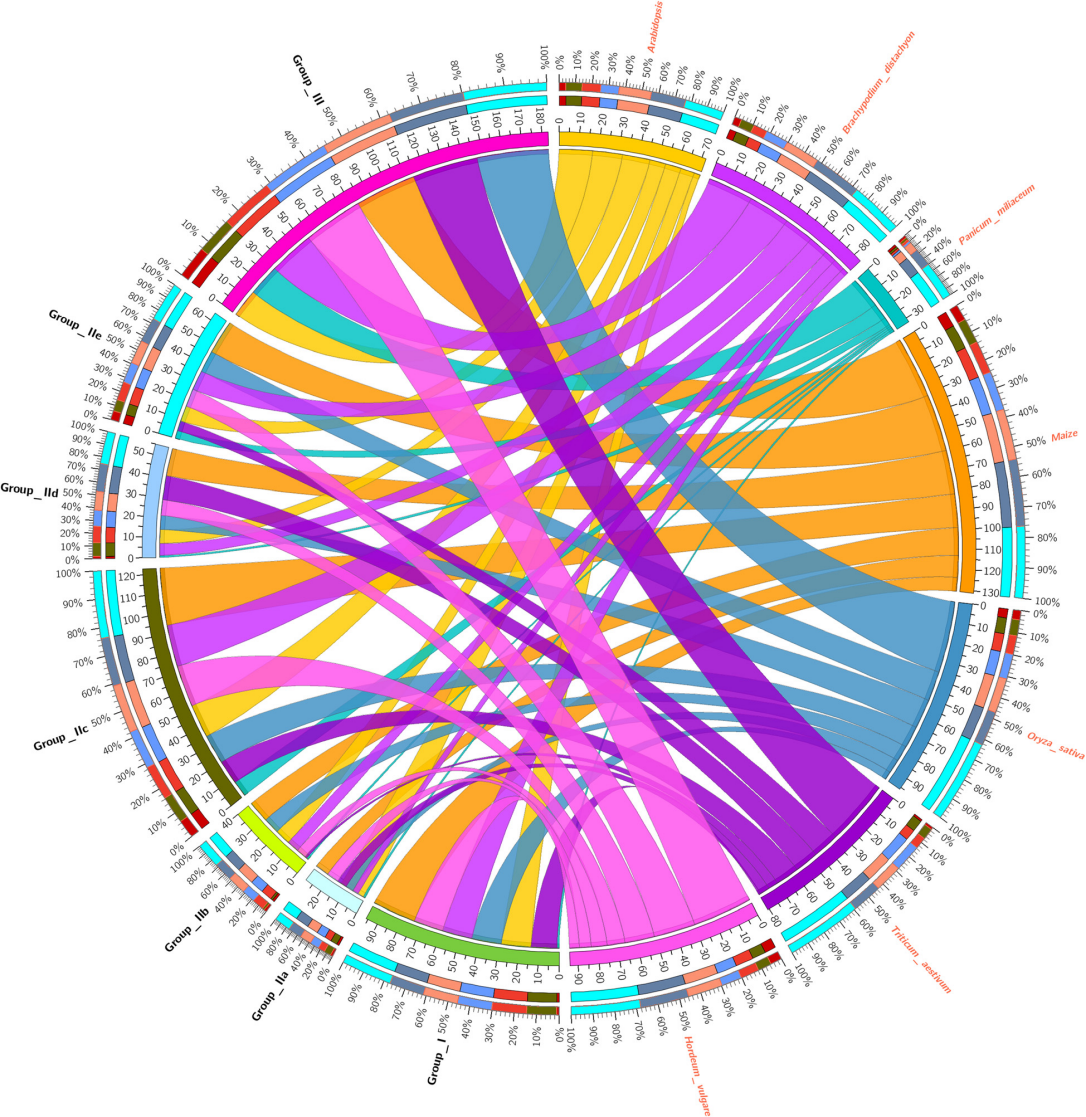
The distribution of WRKY transcription factors from Broomcorn millet, *Arabidopsis thaliana*, *Oryza sativa*, *Brachypodium distachyon*, *Triticum aestivum*, *Hordeum vulgare*, and *Zea mays*

It was reported that variety size of WRKY transcription factor mainly depended on the variations of group III among WRKY genes, and group III of WRKY genes also played a pivotal role in evolutionary relationship [23]. Previous studies have reported that group III was the largest group of WRKY gene family in rice and wheat, which accounted for 38 and 41 %, respectively [26, 27]. while in Arabidopsis, the group II was the largest group, accounting for 24 % [7]. In this study, the group III is also found to be the largest group of WRKY gene family in broomcorn millet, accounting for 50 % of all PmWRKYs, which was consistent with the results of rice and wheat but different from Arabidopsis (Fig. 2).

### Protein structure analysis of PmWRKY

The protein structure of *PmWRKY* was further analyzed. The results showed that 23 PmWRKYs contained highly conserved sequence WRKYGQK. Among them, five PmWRKYs, including *PmWRKY2*, *PmWRKY15*, *PmWRKY23*, *PmWRKY24*, and *PmWRKY28* proteins also have the most common variant sequence WRKYGKK. Adiditionally, four PmWRKYs, including *PmWRKY5*, *PmWRKY6*, *PmWRKY8*, and *PmWRKY20* proteins contained the less common variant sequence WRKYGEK (Additional file 1: Figure S1). These results indicated that nine PmWRKY proteins had a single amino acid variation in their WRKY domain. It was reported that a single amino acid variation for WRKY domain had widely distributed in many species [28, 29]. Previous study demonstrated that five unusual VvWRKY domains possessed variations in grape, and seven unusual TaWRKY domains showed variations in wheat [23].

The WRKY domain is the most vital structural of WRKY proteins, and the usual WRKY domain is WRKYGQK motif, which can interact with the TTGACY core motif to activate downstream genes [7]. It has demonstrated that variance of the WRKYGQK motif could influence the normal activity of DNA binding [30]. When WRKY genes lack the WRKYGQK motif, the WRKYGKK motif may recognize binding sequences excluding the W-box element. For instance, tobacco WRKY protein *NtWRKY12* and soybean WRKY protein *GmWRKY6* cannot bind W-box element, while they can bind to WK-box (TTTTCCAC) [19, 31]. In addition, the variations were also found in zinc-finger motif of PmWRKYs, which was also reported in three VvWRKY proteins. However, the function of variations in zinc-finger motif remains unclear, which had only influence on the classification of the WRKY genes [23].

### Conserved motifs analysis of PmWRKY

**Fig. 3:**
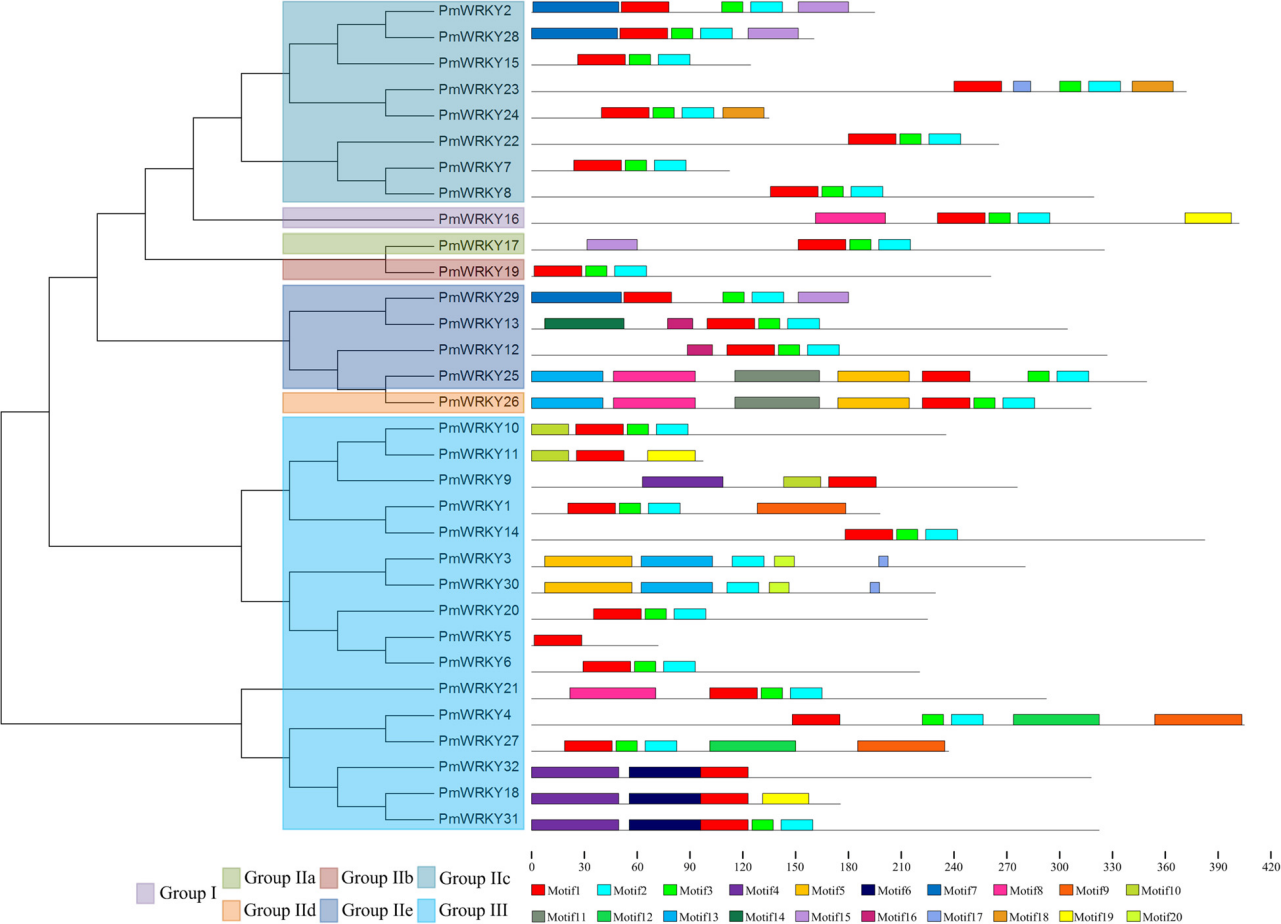
Unrooted phylogenetic tree (*left*) and conserved motifs (*right*) of 32 PmWRKY proteins. The phylogenetic tree was constructed by MEGA4.0. Different color represented various groups. MEME was used to predict motifs and these motifs represented with boxes

MEME program was used to predict the conserved motifs of PmWRKY proteins. A total of 20 motifs were identified to illustrate the WRKY protein structure in broomcorn millet (Fig. 3). Details of 20 conserved motifs were showed in Additional file 2: Figure S2. Results found that the number of motifs in PmWRKYs ranged from 1 to 7, and the length of motifs ranged from 7 to 50 amino acids. In addition, 3 motifs, namely motif 1, 2, and 3 were found in the WRKY domain. The others 17 motifs were found to be located outside the WRKY domain (Additional file 2: Figure S2). Meanwhile, motif 1, 2, and 3 were shared by 26 PmWRKYs, of which motif 1 was shared by *PmWRKY5*, *PmWRKY9*, *PmWRKY11*, *PmWRKY26*, and *PmWRKY32*, while motif 1 and motif 3 were shared by PmWRKY29. Furthermore, three group III members, viz *PmWRKY18*, *PmWRKY31* and *PmWRKY32* shared the motif 4. What is more, motif 8, constituted calmodulin-binding domain (CaMBD), were shared by group I (*PmWRKY16*), IIe (*PmWRKY25*), IId (*PmWRKY26*) and III (*PmWRKY21*) [32]. Additionally, it is generally recognized that WRKYs have a consensus coactivator domain, which was defined as LXXLL [33]. In the present study, it is found that motif 13 have LXXLL-like motif. Two PmWRKY proteins, including *PmWRKY25* and *PmWRKY26* were found to possess motif 8 (Fig. 3 and Additional file 2: Figure S2).

### Homologous gene and functional annotation of PmWRKY proteins in broomcorn millet and rice

Orthologues and paralogues, two types of homologous genes, diverged through speciation in different species or duplication in single specie, respectively. Orthologous genes are widely distributed species and they are generally assumed to perform equivalent biological functions to share key properties of other species [34]. Many methods have been developed to identify the orthologous gene for studying the gene function [35]. Among them, the phylogenetic analysis is one of the most rapid, simple and relative accurate approach to evaluate the orthologs, which has been widely used in different organisms nowadays. As shown in Additional file 3: Table S1, several orthologous genes had been identified. Among these genes, one PmWRKY gene was found to be considered as the homologous genes of several OsWRKYs. For instance, *PmWRKY5* is the homologous genes of *OsWRKY18* and *OsWRKY55.* At the same time, several PmWRKY proteins were also found to have the same homologous gene in rice. For instance, *PmWRKY4* and *PmWRKY27* have the same homologous gene *OsWRKY45*.

**Fig. 4:**
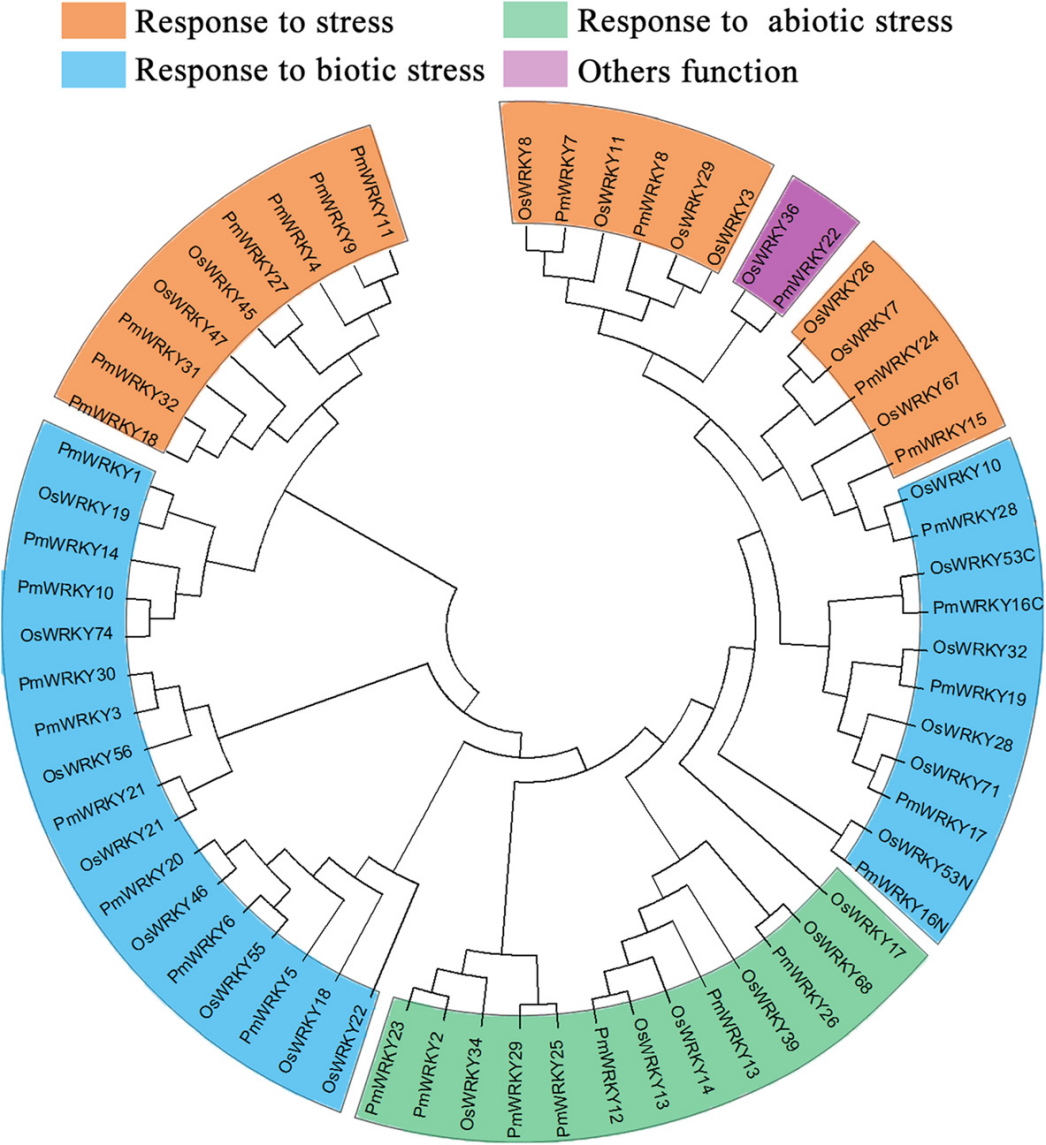
Functional annotation of PmWRKY proteins based on homologous gene of rice. Unrooted phylogenetic tree were constructed for rice and Broomcorn millet proteins to identify homologous gene pairs

It has been widely proved that the function of the given genes could be putatively predicted according to their identified homologous genes. Due to the function of several OsWRKYs had been well studied [18, 36–48], the homologous WRKYs in rice could offer a reference for exploring the potential roles of PmWRKYs. Depending on the function of rice homologs, the function of some PmWRKYs was further putatively analyzed. As shown in Fig. 4, four groups were identified, of which seven *PmWRKY* genes were involved in abiotic stress responses. In addition, 14 PmWRKYs were found to involve in biotic stresses response, and the remaining 11 PmWRKYs were involved in other functions. Previous researches have reported that several WRKY genes had been well described in terms of their important roles in responses to abiotic and biotic stress [49, 50]. It was reported that about 51 % of AtWRKY genes in roots showed significantly high expression levels under salt stress [51], and 57 % of OsWRKY were differentially expressed in response to cold, drought, and salt stress [52] as well as, 70–90 % of VvWRKY genes have significantly differential expressions under various abiotic or biotic stresses [23]. Result showed that the proportion of the WRKYs involving in stresses response in broomcorn millet is similar with other plant.

### Tissue-specific expression patterns of PmWRKY

**Fig. 5:**
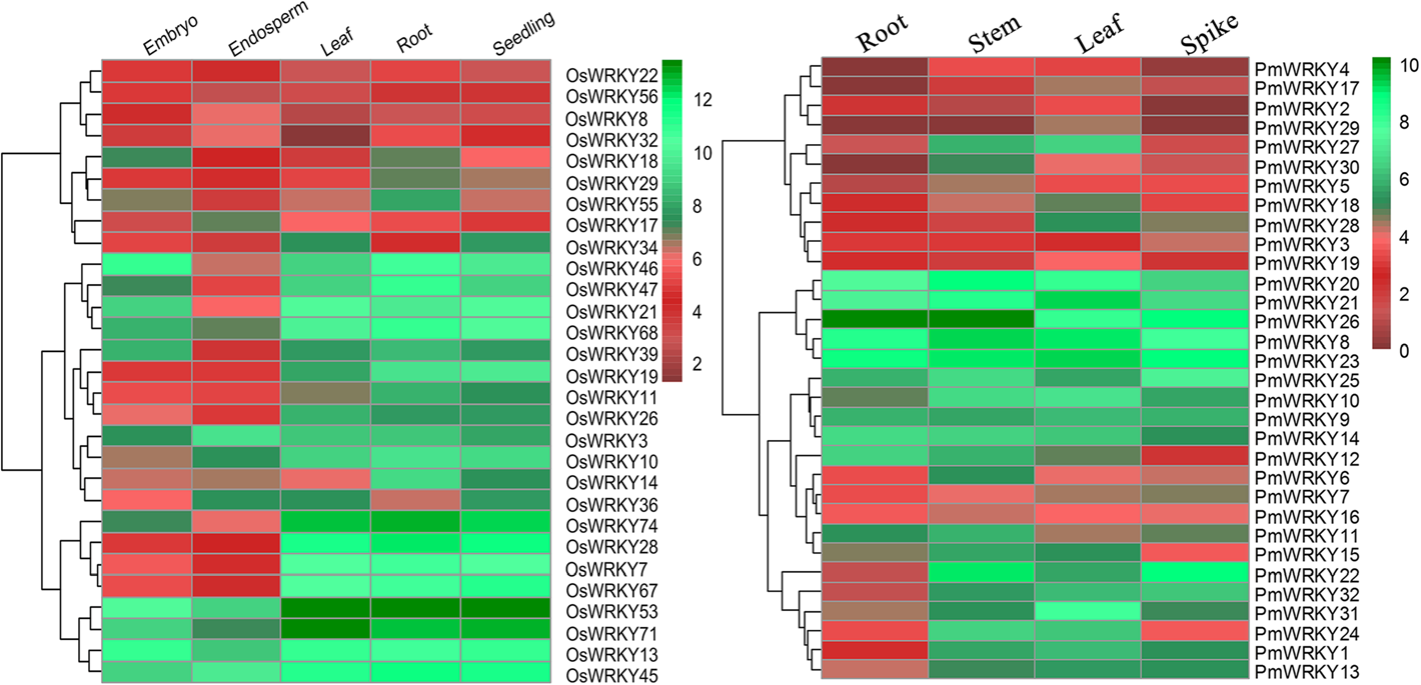
Relative expression level of 32PmWRKY genes (*left*) and 29 homologous gene OsWRKY(*right*) in specific tissues. The result of qRT-PCR was analyzed by TanonGis Tools. Red represents decreased expression level and green represents increased expression level

To explore the tissue specificity of PmWRKY genes, the expression levels of 32 PmWRKY genes were detected by RT-PCR in root, stem, leaf, and spike (Fig. 5 and Additional file 4: Figure S3). Almost one-third of PmWRKY genes represented clear tissue-specific expression. Among them, *PmWRKY3* and *PmWRKY17* had high expression levels in spike and leaf, respectively. The lowest expression of *PmWRKY1* was found in root, while the strongest expressions of *PmWRKY1* were detected in the stem, leaf, and spike. Furthermore, the expression levels of some PmWRKY genes were found to have no significant differences. Additionally, tissue-specific expression patterns of some PmWRKY genes were found to be consistents with their orthologous genes in rice (Additional file 4: Figure S3). *PmWRKY2* represented relatively strongest expression in leaf tissue, and its orthologous gene *OsWRKY34* had highly expression level in the same tissue. Nevertheless, some PmWRKY genes and its orthologous gene appeared to differential expression profiles. It is found that *PmWRKY31* gene had a leaf-specific expression, while its orthologous gene *OsWRKY47* had highly expression levels in root. Totally, more than half of *PmWRKY* genes showed relative consistent expression profiles with their orthologous genes, suggesting that the orthologous genes have similar biological functions with each other (Fig. 5).

### Expression analysis of PmWRKY genes under abiotic stress

**Fig. 6:**
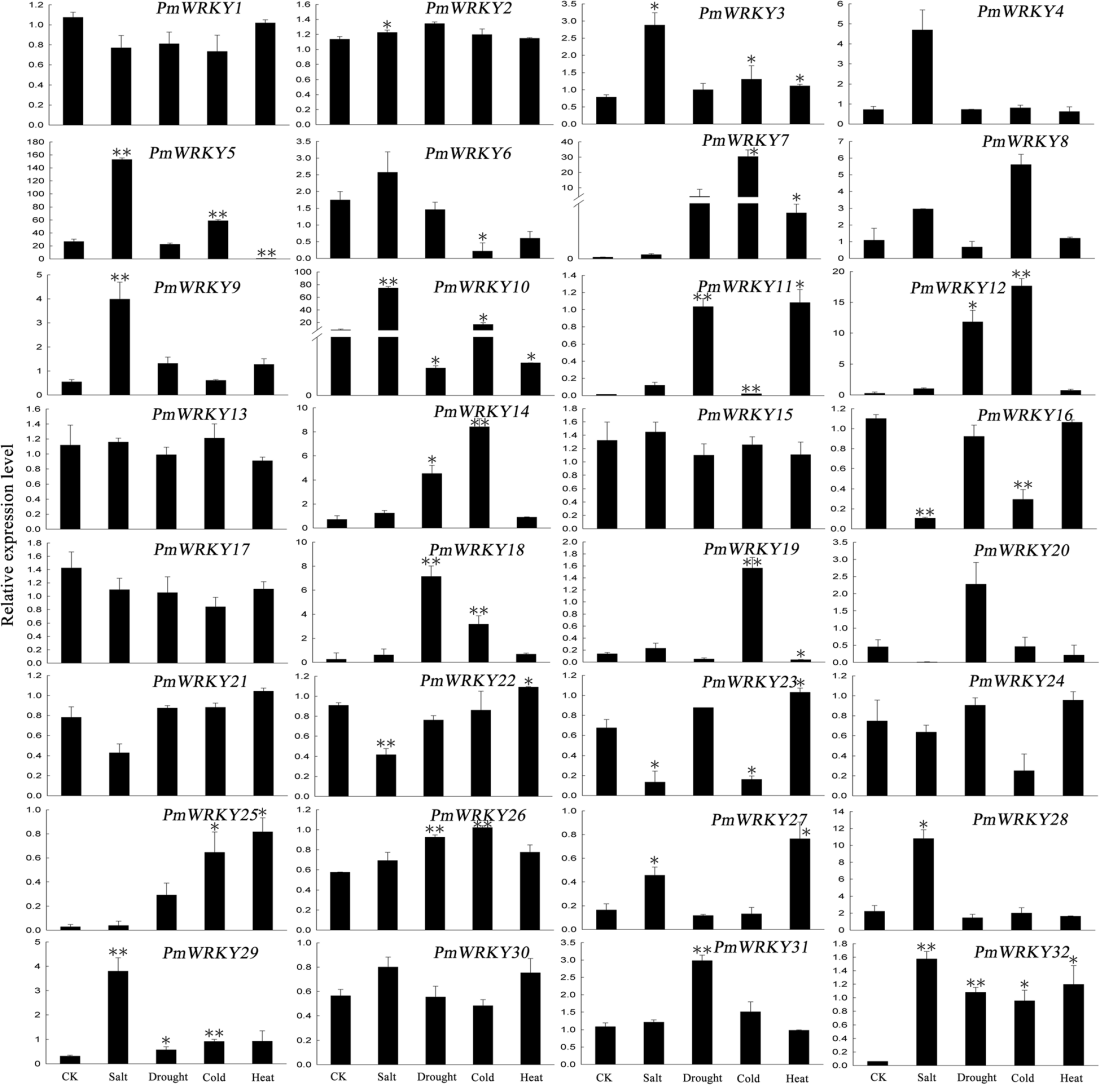
qRT-PCR was used to analyze the expression profiles of 32 PmWRKY genes under salt, drought, cold and heat stress. CK is untreated seedling. Error bars were obtained from three technical replicates. Asterisks reveal the gene significantly up-regulated or down-regulated under abiotic stresses by t-test (*P <0.05, **P <0.01)

In order to verify the function of PmWRKY genes in broomcorn millet and provide the effective informations for future functional analysis, qRT-PCR analysis was used to reveal the differential expressions of each PmWRKYs under different abiotic stresses. The result indicated that 10 and 16 PmWRKY genes were significant induced by drought stress and cold stress treatment, respectively. Additionally, 11 genes were found to be induced by salt and heat stresses. Finally, a total of 22 PmWRKY genes had significantly differential expression under one or more stresses, which could be considered as stress-responsive genes. Interestingly, a diverse range of stress-specific expression profiles of these stress-responsive genes were found. The expression levels of *PmWRKY32* and *PmWRKY11* under stresses conditions were 26-fold and 108-fold higher than that of control condition, respectively. It is incredible that the expression level of *PmWRKY7* was rapidly increased to 216-fold, 1500-fold under drought and cold treatment in comparsion with control group, respectively (Fig. 6). WRKY proteins domain have W box binding site (a four-stranded β-sheet with a zinc-binding pocket) to bind W box [53], which is the key regulatory element involving in stress response. It has reported that 11 OsWRKY genes and 8 TaWRKY genes were induced by salt, drought, cold, and heat stresses [22, 37]. OsWRKY69 was found to specifically bind ABL1 to regulates the rice stress responses [54]. BhWRKY1 were reported to bind W boxes of BhGolS1 under dehydration and ABA stresses [55]. Moreover, some WRKY proteins could interact with promoter elements, such as WK-box [56]. Therefore, these genes may be the invaluable factors in the stress metabolic network of broomcorn millet, and played the important role in abiotic stress response.

Furthermore, it was found that orthologous genes have difference in expression patterns under stress treatment. *PmWRKY25* was found to be up-regulated under salt, drought, cold and heat treatments, while its orthologous gene *OsWRKY17* was down-regulated under these stresses treatments [57]. Furthermore, the expression levels of two PmWRKY genes, including *PmWRKY10* and *PmWRKY32* were the most significant different in all abiotic stresses. Meanwhile, four PmWRKY genes inculding *PmWRKY3*, *PmWRKY5*, *PmWRKY23*, and *PmWRKY29* were rapidly and significantly expressed under one given stress, but these genes have no significant variance in other stresses. The expression level of *PmWRKY23* have significantly up-regulated under heat stress, while it was down-regulated under salt or cold stresses and had no significant variance under drought stress. These results indicated the different PmWRKYs played the different role in regulating stress response, and further investigation of the functions for these PmWRKY genes is necessary. The results reported here provided some insight into the role of PmWRKYs playing in stress response and also provided the candidates for future functional studies.

## Conclusions

In this study, 32 *PmWRKY* genes were identified, which was the first study to investigate the organization and abundance of WRKY in broomcorn millet. The protein structure, conserved motif composition, gene classification and phylogenetic relationship of these PmWRKYs were systematically analyzed and compared. Furthermore, in comparision with rice, orthologous genes analysis was performed to putatively predict the biological function of PmWRKYs. Finally, qRT-PCR analysis was used to investigate the transcriptional profiles in various tissues and under abiotic stresses. Based on the qPCR results, the tissues-specific and stress-responsive PmWRKYs were obtained. This study provide the helpful informations for further investigation of the function of PmWRKYs, and also shed light on the roles of *WRKY* genes playing in regulating development and physiological processes in this important stress-tolerance crops.

## Methods

### WRKY identification

A total of 211 original ESTs of broomcorn millet were obtained from the NCBI dbEST database (http://www.ncbi.nlm.nih.gov/dbEST/). The plant tissues of two Broomcorn millet cultivars (Yumi No.2 and Yumi No.3), including leaves, stems, roots, shoots, and spikes of different grown stages, were separately selected from three different plants for RNA extraction. The equal quantity RNA of each sample from the same variety was pooled and used for RNA-seq. Then, the public available ESTs together with the unigenes obtained from RNA-seq were used to predict WRKY genes. The WRKY cDNA sequences of *Setaria italic* were obtained from the Foxtail millet Transcription Factor Database (http://59.163.192.91/FmTFDb/index.html), and then these sequences were used to search the EST database through BLAST search with an e-value cut-off <10^−5^. The best hits were extracted as the EST representing PmWRKY. After manual curation, these ESTs were clustered and assembled by CAP3 tool and then obtained contigs and singletons were checked using NCBI-CDD search (http://www.ncbi.nlm.nih.gov/Structure/cdd/wrpsb.cgi) to identify the conserved protein domain. The sequences contained the complete WRKY domain was further used to BLAST against the FmTFDb database to remove redundant. Finally, a total of 32 putative PmWRKY genes were obtained and termed as *PmWRKY1* to *PmWRKY32*.

### Multiple sequence alignment, conserved motifs identification and Phylogenetic analysis

The *Arabidopsis thaliana* WRKY genes were obtained from DATF, while those of rice and maize were obtained from previous studies [24, 58]. To investigate the evolutionary relationships among these WRKYs, the predicted amino acid sequences of the WRKY genes of broomcorn millet and other species were aligned using ClutsalX1.83 program and then sequence identity and similarity were calculated. The phylogenetic tree was constructed using MEGA6.0 and neighbor-joining (NJ) method were adopted by 1,000 bootstrap replications [7]. Motifs of PmWRKYs were determined by using the MEME program (http://meme-suite.org/tools/meme) and a schematic diagram of amino acid motifs of each PmWRKY gene was drawn accordingly.

### Plant materials and abiotic stress treatments

Broomcorn millet cultivar Yumi No.3 was kindly provided by Prof. Baili Feng in this study. Seeds were planted in flowerpots and filled with a mixture of soil and sand (at a ratio of 1:1, v/v), and then it were adequately watered and grown in growth room under the condition of 22 °C, 16 h photoperiod (10,000 lux) and 20 °C, 8 h dark period. Leafs and roots were harvested at the given time after sowing: seedling stage (the 2^th^ week), jointing stage (the 4^th^ week), booting stage (the 6^th^ week), filling stage (the 8^th^ week) and mature stage (the 14^th^ week). Stems were harvested at jointing stage (the 4^th^ week), booting stage (the 6^th^ week), filling stage (the 8^th^ week) and mature stage (the 14^th^ week). Spikes were harvested at mature stage (the 14^th^ week). Meantime, in the fourth weeks, old whole seedlings of Yumi No.3 were treated by 200 mM NaCl, 19.2 % PEG, 4 °C or 38 °C for 24 h, which represented salt, drought, cold and heat treatment respectively. Then, these plants materials were collected and immediately frozen in liquid nitrogen until RNA extraction.

### RT-PCR and qRT-PCR analysis

Total RNA from collected samples were isolated and performed with three independent experiments using the plant RNA isolation kit (Omega BioTek, USA). Concentration of the RNA was measured, and then equivalent mixed of three independent RNA for each sample to ensure that each reverse transcriptase reactions contained an equal amount of RNA from three independent seedlings. 1 μg high-quality RNA was used to synthesize the complementary DNA by the cDNA amplification kit (Vazyme, China) for downstream use. Primer Premier 5.0 was used to designed the primers for each PmWRKY gene and actin as reference gene [59] (Additional file 5: Table S2). The thermal profile for RT-PCR was as follows: 30 s at 94 °C, 35 cycles of 5 s at 94 °C, and 30 s at 58–60 °C, last 72 °C for 1 min. PCR products were checked by 1.0 % (w/v) agarose gel electrophoresis. The expression levels of specific tissues were analyzed from RT-PCR data with using the tanon Gis tool. qRT-PCR were performed using ABI 7300 real-time PCR system (Applied Biosystems, Grand Island, NY) based on SYBR Green II method. The 20 μl qRT-PCR mixtures contained 10 μl SYBR Premix buffer, 0.8 μl each of the primers (10 μM), 0.4 μl ROX Reference Dye (10 μmol/L), 2 μl cDNA and 6.8 μl PCRgrade water. The thermal profile for qRT-PCR was as follows: 30 s at 94 °C, 40 cycles of 5 s at 94 °C, and 30 s at 60 °C, last 72 °C for 1 min. Each reaction was run in triplicate to obtain the average value and △Ct method was applied for the analysis gene expression profiles under abiotic stresses, and no template reactions were used as negative controls.

## Additional files

Additional file 1: Figure S1. Multiple-sequence alignment of the WRKY protein domain from PmWRKYs and OsWRKYs. Conserved amino acids were indicated by blank background, conserved WRKY domains and zinc-finger motifs were indicated by red box. (DOC 1365 kb) Additional file 2: Figure S2. Sequence logos of PmWRKY domain. The PmWRKY proteins domain submitted to MEME server. The total height of stack was used to shows ‘information content’ of that position in the motif. Height of letters in stack suggests probability of each amino acids at that position. (DOC 558 kb) Additional file 3: Table S1. The identified PmWRKY proteins in Broomcorn millet and their putative orthologous gene in rice. (DOC 47 kb) Additional file 4: Figure S3. Expression level of 32 PmWRKY genes in different tissues. Actin was an internal reference gene. (DOC 797 kb) Additional file 5: Table S2. Primers of 32 PmWRKY genes. (DOC 54 kb)
