# Supplementary material for: Transcriptome-wide identification and expression profiles of the WRKY transcription factor family in Broomcorn millet (Panicum miliaceum L.)
Source: BMC Genomics. 2016 May 10;17:343. doi: 10.1186/s12864-016-2677-3 (PMC4862231; doi:10.1186/s12864-016-2677-3)
Supplement: Additional file 1: Figure S1. — Multiple-sequence alignment of the WRKY protein domain from PmWRKYs and OsWRKYs. Conserved amino acids were indicated by blank background, conserved WRKY domains and zinc-finger motifs were indicated by red box. (DOC 1365 kb) [file 12864_2016_2677_MOESM1_ESM.doc]

**Additional file 3: Figure S1**


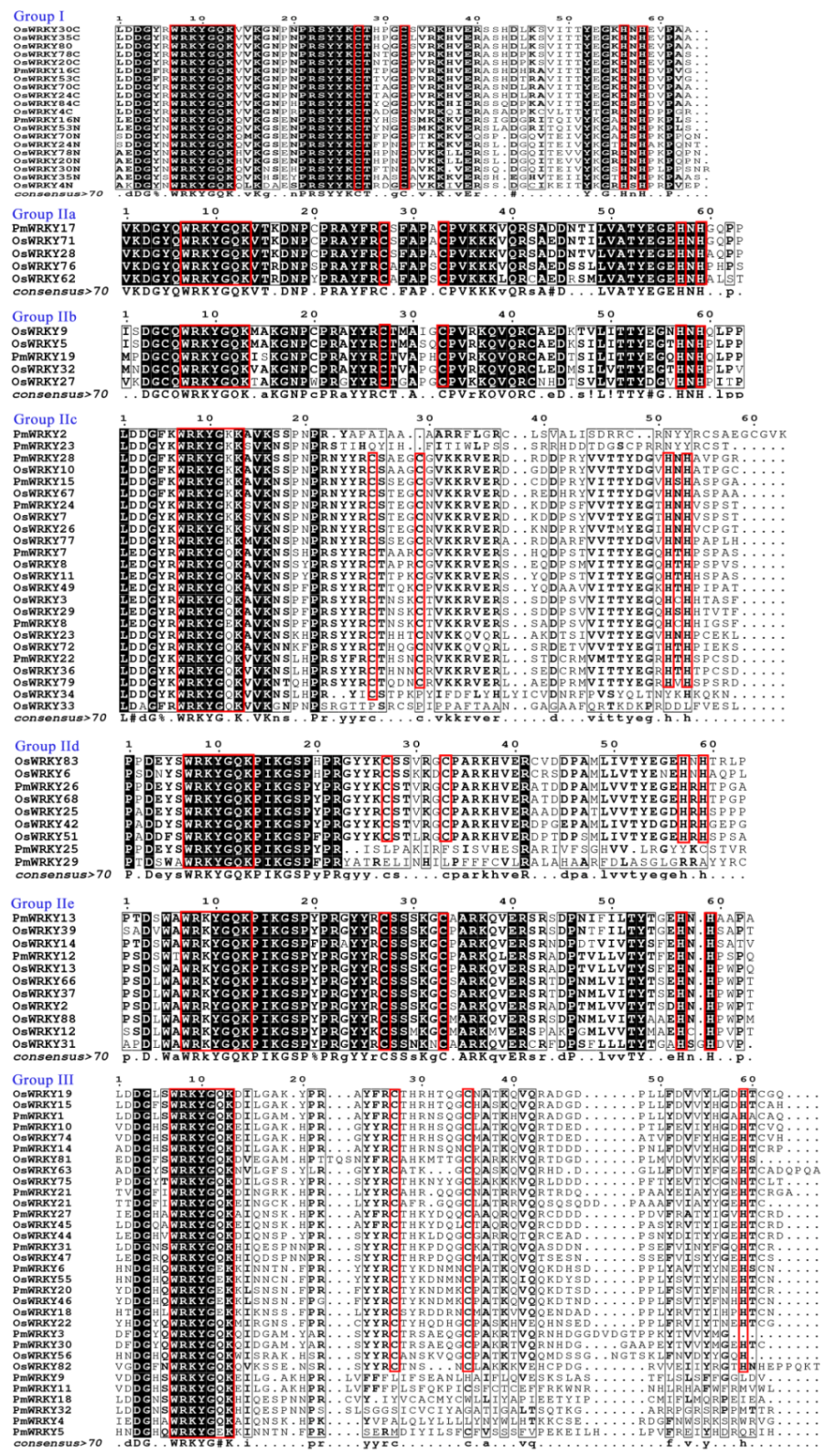


**Figure S1** Multiple-sequence alignment of the WRKY protein domain from PmWRKYs and OsWRKYs. Conserved amino acids were indicated by blank background, conserved WRKY domains and zinc-finger motifs were indicated by red box.
