# Supplementary material for: Transcriptome-wide identification and expression profiles of the WRKY transcription factor family in Broomcorn millet (Panicum miliaceum L.)
Source: BMC Genomics. 2016 May 10;17:343. doi: 10.1186/s12864-016-2677-3 (PMC4862231; doi:10.1186/s12864-016-2677-3)
Supplement: Additional file 2: Figure S2. — Sequence logos of PmWRKY domain. The PmWRKY proteins domain submitted to MEME server. The total height of stack was used to shows ‘information content’ of that position in the motif. Height of letters in stack suggests probability of each amino acids at that position. (DOC 558 kb) [file 12864_2016_2677_MOESM2_ESM.doc]

**Additional file 4: Figure S2**


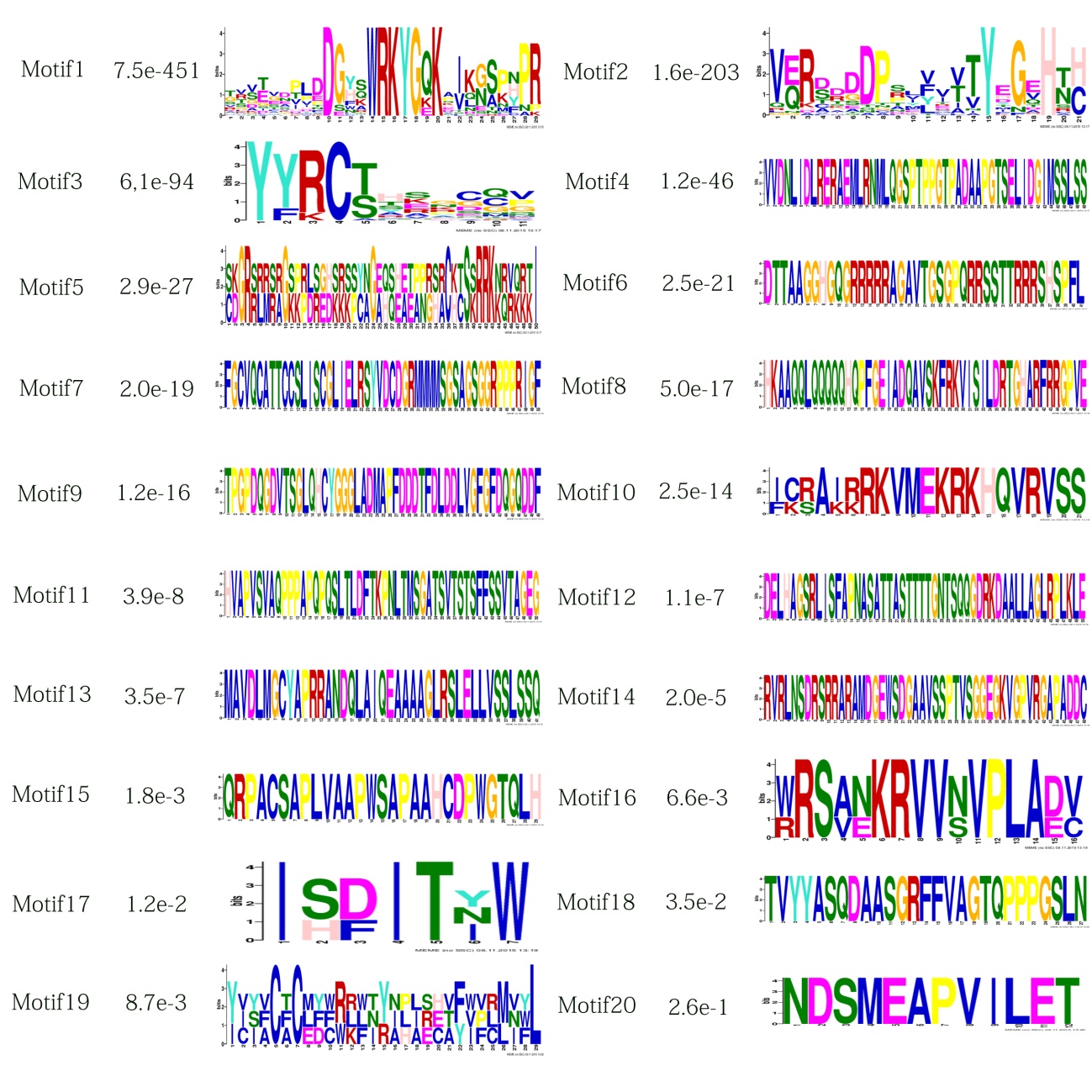


**Figure S2** Sequence logos of PmWRKY domain. The PmWRKY proteins domain submitted to MEME server. The total height of stack was used to shows ‘information content’’ of that position in the motif. Height of letters in stack suggests probability of each amino acids at that position.
