# Supplementary material for: Transcriptome-wide identification and expression profiles of the WRKY transcription factor family in Broomcorn millet (Panicum miliaceum L.)
Source: BMC Genomics. 2016 May 10;17:343. doi: 10.1186/s12864-016-2677-3 (PMC4862231; doi:10.1186/s12864-016-2677-3)
Supplement: Additional file 3: Table S1. — The identified PmWRKY proteins in Broomcorn millet and their putative orthologous gene in rice. (DOC 47 kb) [file 12864_2016_2677_MOESM3_ESM.doc]

**Additional file 1: Table S1** The identified PmWRKY proteins in Broomcorn millet and their putative orthologous gene in rice.

| PmWRKY proteins | Gene ID | Orthologs Os WRKY proteins | Gene ID |
| --- | --- | --- | --- |
| PmWRKY1 | KU058619 | OsWRKY19 | AK108389 |
| PmWRKY2 | KU058606 | OsWRKY33, OsWRKY34 | DAA05098.1,AK072906 |
| PmWRKY3 | KU058620 | OsWRKY22, OsWRKY75 | NP_001147816.1, DAA05140.1 |
| PmWRKY4 | KU058621 | OsWRKY44, OsWRKY45 | AK105244, AK066255 |
| PmWRKY5 | KU058622 | OsWRKY18, OsWRKY55 | DAA05083.1,AK101653 |
| PmWRKY6 | KU058623 | OsWRKY55 | AK101653 |
| PmWRKY7 | KU058607 | OsWRKY8, OsWRKY11 | AY341857, AY341856 |
| PmWRKY8 | KU058608 | OsWRKY3, OsWRKY29 | AY341859, AY341858 |
| PmWRKY9 | KU058624 | OsWRKY74 | AK065265 |
| PmWRKY10 | KU058625 | OsWRKY74 | AK065265 |
| PmWRKY11 | KU058626 | OsWRKY74 | AK065265 |
| PmWRKY12 | KU058617 | OsWRKY13, OsWRKY14 | AK067329, AK109770 |
| PmWRKY13 | KU058618 | OsWRKY31, OsWRKY39 | DAA05096.1,AK066775 |
| PmWRKY14 | KU058627 | OsWRKY74 | AK065265 |
| PmWRKY15 | KU058609 | OsWRKY67 | AK066252 |
| PmWRKY16 | KU058603 | OsWRKY53 | DAA05118.1 |
| PmWRKY17 | KU058604 | OsWRKY28, OsWRKY71 | AK106282, DAA05136.1 |
| PmWRKY18 | KU058628 | OsWRKY44, OsWRKY47 | AK105244, AK110900 |
| PmWRKY19 | KU058605 | OsWRKY32 | DAA05097.1 |
| PmWRKY20 | KU058629 | OsWRKY46 | AK073243 |
| PmWRKY21 | KU058630 | OsWRKY21, OsWRKY56 | AK108657, AK102093 |
| PmWRKY22 | KU058610 | OsWRKY36 | AK073695 |
| PmWRKY23 | KU058611 | OsWRKY33, OsWRKY34 | DAA05098.1, AK072906 |
| PmWRKY24 | KU058612 | OsWRKY7, OsWRKY26 | DAA05072.1, AK108555 |
| PmWRKY25 | KU058614 | OsWRKY17, OsWRKY34 | AK110625, AK072906 |
| PmWRKY26 | KU058615 | OsWRKY68 | AK061266 |
| PmWRKY27 | KU058631 | OsWRKY44, OsWRKY45 | AK105244, AK066255 |
| PmWRKY28 | KU058613 | OsWRKY10 | AK109578 |
| PmWRKY29 | KU058616 | OsWRKY17, OsWRKY34 | AK110625, AK072906 |
| PmWRKY30 | KU058632 | OsWRKY22, OsWRKY75 | NP_001147816.1, DAA05140.1 |
| PmWRKY31 | KU058633 | OsWRKY44, OsWRKY47 | AK105244, AK110900 |
| PmWRKY32 | KU058634 | OsWRKY44, OsWRKY47 | AK105244, AK110900 |
