# Supplementary material for: Transcriptome-wide identification and expression profiles of the WRKY transcription factor family in Broomcorn millet (Panicum miliaceum L.)
Source: BMC Genomics. 2016 May 10;17:343. doi: 10.1186/s12864-016-2677-3 (PMC4862231; doi:10.1186/s12864-016-2677-3)
Supplement: Additional file 4: Figure S3. — Expression level of 32 PmWRKY genes in different tissues. Actin was an internal reference gene. (DOC 797 kb) [file 12864_2016_2677_MOESM4_ESM.doc]

**Additional file 5: Figure S3**


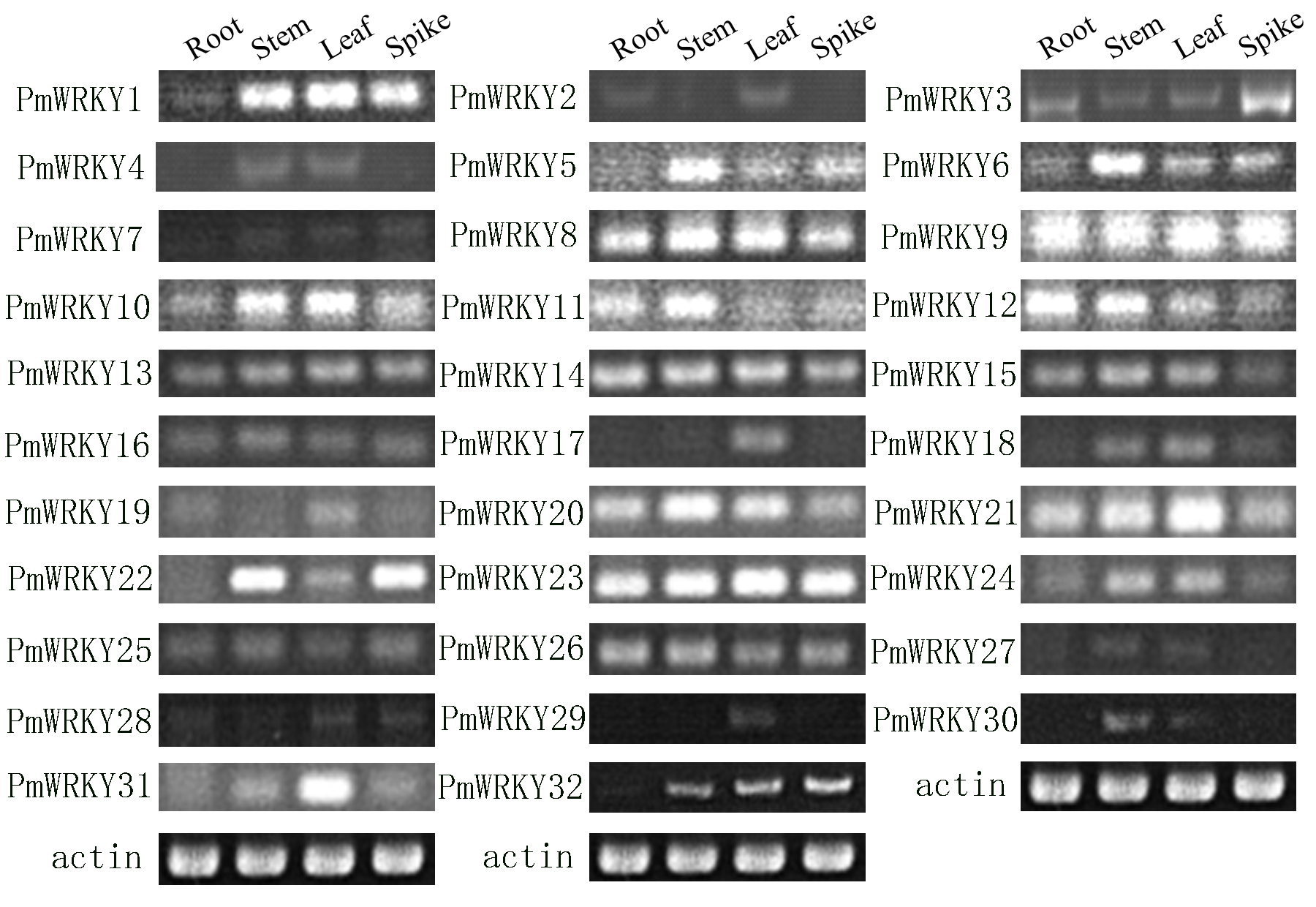


**Figure S3** Expression level of 32 PmWRKY genes in different tissues. Actin was an internal reference gene.
