# Supplementary material for: Transcriptome-wide identification and expression profiles of the WRKY transcription factor family in Broomcorn millet (Panicum miliaceum L.)
Source: BMC Genomics. 2016 May 10;17:343. doi: 10.1186/s12864-016-2677-3 (PMC4862231; doi:10.1186/s12864-016-2677-3)
Supplement: Additional file 5: Table S2. — Primers of 32 PmWRKY genes. (DOC 54 kb) [file 12864_2016_2677_MOESM5_ESM.doc]

**Additional file 2: Table S2** Primers of 32 PmWRKY genes

| PmWRKY gene | Gene ID | Primers | | Product (bp) |
| --- | --- | --- | --- | --- |
|  |  | Forward primer(5’-3’) | Reverse primer(5’-3’) |  |
| PmWRKY1 | KU058619 | GACTTGTACGCGGCCAATTAG | GACAAGGCCATAGCACCCAAC | 155 |
| PmWRKY2 | KU058606 | AAGAAGGCGGTCAAGAGCAG | AATGAGCGCAACGGAAAGG | 98 |
| PmWRKY3 | KU058620 | CGGATGGGTGGACGATTGAC | CCGGCTACTCCGACAAGACAG | 111 |
| PmWRKY4 | KU058621 | CTCCGCAGCCATAAATACCC | AGCTCGATCTCGCACTCCC | 130 |
| PmWRKY5 | KU058622 | TGGAGGAAGTACGGCGAGAAG | TTTCAGGAACAAACGAGGACG | 112 |
| PmWRKY6 | KU058623 | CGCATTTCTTGATTTCTTCTTCC | TCGCCGTACTTCCTCCATTG | 152 |
| PmWRKY7 | KU058607 | GCGACCTGTCGTCCTTGATG | GCCTTCTGGCCGTACTTGC | 188 |
| PmWRKY8 | KU058608 | GATATGGTGCCTCATGGAGTAAG | TAATGCCGCAGGAGATAGAGAAC | 145 |
| PmWRKY9 | KU058624 | TTTTGGAGGTTTGGATGTTGTG | CACCAGAACAAAATTAGCACAAAC | 135 |
| PmWRKY10 | KU058625 | GGAGGAAGTACGGCCAGAAGG | GCCTCTGAACGCAGGTGTG | 184 |
| PmWRKY11 | KU058626 | ATTCGCAGGAAGGTGATGGAG | ACTGCAGATTGGCTTCTGAAAAG | 188 |
| PmWRKY12 | KU058617 | CCGTGCTGCTCGTCACCTAC | CCTGCTCCTGTTCTTGCTCC | 215 |
| PmWRKY13 | KU058618 | TACGTGCCGTCCTTGTAGATG | AAAGGCTATAAAGGTTGGATTGG | 184 |
| PmWRKY14 | KU058627 | GGAGAGGAGCAGAGGACTAGAGG | TGATTCCTTTTCTCTTGCTGACTG | 187 |
| PmWRKY15 | KU058609 | GTGGTGCAAGTGCAAGGACAG | AGGAGTAGGACGGCGAGTAGG | 118 |
| PmWRKY16 | KU058603 | GTACGTAATTGCTTGCTTCTGCC | CCTCCTTCCTGAGGAAAATCAATAG | 126 |
| PmWRKY17 | KU058604 | GAGGGCGAGCACAACCAC | AACGCTCATCGCAGCCAC | 191 |
| PmWRKY18 | KU058628 | TCTCGATCCGCCTCTATCTCC | AACTCGTCGGTGACATTCGG | 107 |
| PmWRKY19 | KU058605 | CGGGAGCTGGTGGTTGTG | GAGCAGATGCCCGATGGTT | 192 |
| PmWRKY20 | KU058629 | GGCAGGGCACTTCATGTCG | TGGACCTCGGACACTTACGC | 135 |
| PmWRKY21 | KU058630 | CGACGGCACATCCAAATCTAC | CTCCACCTCGCTGGTCTCC | 98 |
| PmWRKY22 | KU058610 | GCAAGTTCCCATCAAGTCTACCC | CCGACTGCTTCAGCTCCTTC | 195 |
| PmWRKY23 | KU058611 | TGAACATCGGACAAAACCTCTG | TCACTTGTTCCCACGAGCATC | 123 |
| PmWRKY24 | KU058612 | CTCCTCCTCCGACCTTACCC | TTTTGACCAGTCGGTTCGTG | 118 |
| PmWRKY25 | KU058614 | ACGTCACCACCAGCATCGC | TCGTTCTCAGGGGCTACTACAAG | 101 |
| PmWRKY26 | KU058615 | TCGTACGTCACCACCAGCATC | GAGAAGGAAAAACCGTGTGAAGAG | 114 |
| PmWRKY27 | KU058631 | CAAAACTCCAAGCACCCAAAG | ATGTAGGTTGCCCTGAAGACG | 121 |
| PmWRKY28 | KU058613 | GAGCTACGTGGATTGTGACGG | GGGTAGTAGTTCCTCGGGTTGG | 186 |
| PmWRKY29 | KU058616 | CTTCCTTTCTTCTTCTGCGTTC | GGGCACCCTTTGTTGCTG | 116 |
| PmWRKY30 | KU058632 | TCCCTGTTACCAGCACCAATAAC | GTCGTAGTCATCGACGACGTAATC | 184 |
| PmWRKY31 | KU058633 | ACCTGCCTGGTCGCCTTG | GGCAACTCATGGAGAAAGTACGG | 115 |
| PmWRKY32 | KU058634 | ATATACATACGCAGATAGATCCGC | AGCAGCACCACCAGGAGAAG | 163 |
| actin | -^a^ | ACCGAAGCCCCTCTTAACCC | GTATGGCTGACACCATCACC | 171 |

^a^ According to
